# Benzene Exposure and Cancer Risk from Commercial Gasoline Station Fueling Events Using a Novel Self-Sampling Protocol

**DOI:** 10.3390/ijerph18041872

**Published:** 2021-02-15

**Authors:** Andrew N. Patton, Misti Levy-Zamora, Mary Fox, Kirsten Koehler

**Affiliations:** 1 Department of Environmental Health and Engineering, Johns Hopkins Bloomberg School of Public Health, 615 N. Wolfe St., Baltimore, MD 21205, USA; andrew.patton@jhu.edu (A.N.P.); misti.levy.zamora@jhu.edu (M.L.-Z.); 2 CARTEEH (Centers for Advancing Research in Transportation Emissions, Energy, and Health), Texas Transportation Institute, 624 N. Broadway, Baltimore, MD 21205, USA; mfox9@jhu.edu; 3 Department of Health Policy and Management and Risk Sciences and Public Policy Institute, Johns Hopkins Bloomberg School of Public Health, 624 N. Broadway, Baltimore, MD 21205, USA

**Keywords:** gasoline, benzene, TVOC, exposure assessment, probabilistic risk assessment

## Abstract

Tens of millions of individuals go to gasoline stations on a daily basis in the United States. One of the constituents of gasoline is benzene, a Group 1 carcinogen that has been strongly linked to both occupational and non-occupational leukemias. While benzene content in gasoline is federally regulated, there is approximately a thirty-year data gap in United States research on benzene exposures from pumping gasoline. Using a novel self-sampling protocol with whole air canisters, we conducted a gasoline pumping exposure assessment for benzene, toluene, ethylbenzene, and xylene (BTEX) on Baltimore, MD consumers. Geometric mean exposures (geometric standard deviations) were 3.2 (2.7) ppb,9.5 (3.5) ppb, 2.0 (2.8) ppb, and 7.3 (3.0) ppb, respectively, on 32 samples. Using the benzene exposures, we conducted consumer and occupational probabilistic risk assessments and contextualized the risk with ambient benzene exposure risk. We found that the consumer scenarios did not approach the 1:1,000,000 excess risk management threshold and that the occupational scenario did not exceed the 1:10,000 excess risk management threshold. Further, in all Monte Carlo trials, the ambient risk from benzene exposure exceeded that of pumping risk for consumers, but that in approximately 30% of occupational trials, the pumping risk exceeded the ambient risk.

## 1. Introduction

According to the National Association of Convenience Stores, in 2019, there were approximately 129,000 convenience store gasoline stations and mass merchandising gasoline stations in the United States, accounting for 96% of all commercial gasoline sold [1,2]. There were approximately 40 million fill-ups per day at these gasoline stations as of 2012 [3]. The main form of gasoline sold is automotive gasoline, the primary fuel for internal combustion engines found in non-diesel cars, motorcycles, non-diesel trucks, and other small engines [4]. Gasoline is a complex, non-uniform mixture comprised of a variety of alkanes, alkenes, isoalkanes, cycloalkanes, cycloalkenes, and aromatics. Many blends also contain performance-enhancing additives [5]. The exact ratios of these compounds vary by manufacturer and location, and even from batch to batch, depending on factors, such as the source of the crude oil, the refining process used in its production, and the product specifications [4,5]. Since the Clean Air Act Amendments of 1990, gasoline frequently contains ethanol in addition to petroleum products. The two most common mixtures in the United States are 10% ethanol/90% gasoline (E10) and 15% ethanol/85% gasoline (E15) [6]. Approximately 95% of gasoline sold in the United States is E10 [6]. However, the amount of ethanol used in blending can vary substantially, with maximums nearing 85% ethanol, primarily used outside of the United States [6].

Gasoline is a known human and animal carcinogen based on the toxicity of its components [4,5]. Amongst the constituents of gasoline, benzene has the strongest body of evidence supporting its carcinogenicity (leukemias) in occupational and non-occupational settings, and the Environmental Protection Agency (EPA), U.S. Department of Health and Human Services, and International Agency for Research on Cancer (IARC), all identify benzene as a human carcinogen [5,7,8]. There is epidemiological and toxicological evidence that excess benzene exposure can result in the development of acute myeloid leukemia (AML) in humans [9,10,11,12]. Other leukemias, including acute nonlymphocytic leukemia (ANLL) and myelodysplastic syndrome (MDS), have also been found to be associated with elevated benzene exposure [7,13]. Benzene content in gasoline is federally regulated, with any refineries or importers required to average less than or equal to 0.62% benzene by volume [14]. Generally, gasoline in the United States is likely to contain 0.5%–2.0% benzene by volume [14,15]. Additionally, in terms of non-occupational exposures, the National Air Toxics Assessment (NATA) ambient air pollution monitoring includes benzene as a ‘national cancer risk contributor’ and provides excess cancer risk associated with that ambient benzene exposure [16,17].

Benzene exposure has been extensively studied in both upstream (petroleum extraction and production) and downstream (refining and marketing) settings [18,19]. However, there is little information regarding potential exposures to the gasoline station consumer, a population of millions of individuals per day in the United States. According to the Agency for Toxic Substances and Disease Registry (ATSDR), non-occupational exposures to gasoline occur as a result of customers using the gasoline pumps and inhaling any volatilized part of the gasoline mixture [4]. The bulk of the studies and samples associated with consumers filling their own vehicles occurred in the 1980s and 1990s and were conducted by consulting firms or industrial sources [18,20,21]. However, there were minimal details provided on sampling methodologies and procedures [18,20,21]. Additionally, of the studies that were conducted in other countries (e.g., Singapore, Italy, England), approximately five percent of the mean benzene concentrations were greater than 2.5 ppm for short-duration, consumer-focused measurements, the short-term occupational exposure limit issued by the American Conference of Governmental Industrial Hygienists [20,22,23]. However, studies conducted in Europe in the 2000s indicate significantly reduced exposures compared to the 1980s and 1990s [20,22,23]. In addition to consumers, there are approximately 21,000 gasoline service station attendants across the country as of 2019 who may also pump gasoline as part of their job description [24]. Furthermore, in New Jersey and Oregon, there are nearly 5000 pump attendants who are legally required to pump gas for customers (the majority of whom reside in New Jersey) [25,26]. 

We conducted an exposure assessment for consumers to characterize benzene and associated volatile organic compounds exposures associated with filling their gas tank using a novel self-sampling protocol that let the participants choose the time, place, and duration of the sampling allowing for a realistic amount of variation in consumer behavior and gasoline station factors. In addition, the exposure assessment was used to inform a consumer risk assessment for gasoline station filling. The risk assessment was extended to an occupational setting by developing worker exposure scenarios to estimate excess risk values for gasoline service station attendants and pump attendants. Lastly, the risk assessment results for the consumer and occupational exposure scenarios were compared to the risk values provided by NATA in order to contextualize the risk from gasoline station benzene exposures with the risk from ambient benzene exposures.

## 2. Materials and Methods

**Figure 1:**
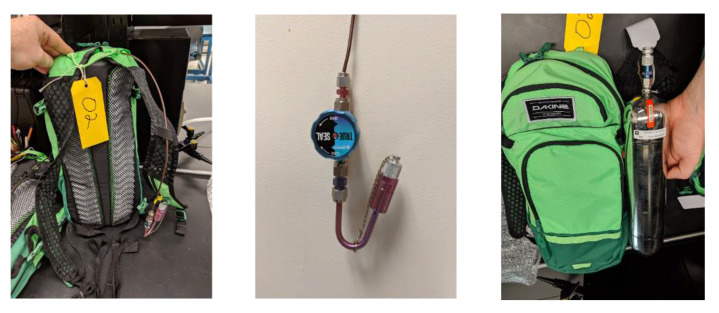
Consumer sampling backpack containing an evacuated steel canister, sampling line, flow regulator and start/stop knob, and MSR climate monitor.

The study participants consisted of 34 Baltimore, Maryland area consumers who were aged 18 or over, English speaking, literate, had a valid driver’s license, had access to a working gasoline-powered vehicle, and were able to fill their vehicle with at least five gallons of gasoline. Active smokers and pregnant or nursing women were excluded. While 100 participants were planned, COVID-19 related shutdowns on research limited the sample size to 34 individuals. At the time of consent into the study, each consumer was provided with a backpack (Figure 1) containing sampling equipment, demographic surveys, and fill-up specific questionnaires including questions, such as ‘How many times per month do you pump gas?’, ‘What make and model of car do you drive?’, and similar questions on related topics. The study was approved by the Johns Hopkins Institutional Review Board (#00008731).

The consumer sampling was conducted using a backpack containing sampling equipment and electronics. The air sampling equipment was comprised of a 1.0 L MiniCan (Entech Instruments, Simi Valley, CA, USA) Silonite lined passivated steel canister evacuated to −30.00 mmHg and an attached Silonite lined steel sampling line with a flow regulator, open/close knob, and screw cap. Once opened, the canisters draw in whole air at 0.167 L/minute, for a six-minute operational limit. However, due to a loose gasket seal on the inlet valve with the first ten canisters used, a revised gasketless valve design was implemented for all other canisters. The flow rate, pressure, and canisters were otherwise identical. The backpack also contained an MSR 145 Data Logger (MSR Electronics GmbH, Seuzach, Switzerland) with temperature, relative humidity, and light sensors recording data on one-second intervals. Chubb Environmental Health Laboratory (Cromwell, CT, USA) provided the canisters and designed and provided the custom sampling lines. Prior to providing each consumer with a backpack, each line was cleared with a clean vacuum canister and each sampling canister had its vacuum measured and recorded with an electronic pressure gauge.

Using the backpack from Figure 1, each consumer was directed to drive to the gas station of their choice (unknown to study staff), open the sampling line and cap, exit their vehicle, and pump gas as they normally would, enter their vehicle when finished, and then close the sampling line and cap. Participants were also instructed to remain near their vehicles while filling. The sampling backpacks were returned to study staff within 24 h. Upon return of each backpack, the canisters were checked to verify use (i.e., change in canister vacuum pressure). No other assessment of protocol adherence was performed. Personal sampling was conducted from August 2019 through March 2020, with sampling stopping due to COVID-19 related shutdowns. Following collection, all canisters were measured for final pressure and returned to Chubb Environmental Health Laboratory (Cromwell, CT, USA). Canisters were analyzed for BTEX (benzene, toluene, ethylbenzene, xylene) via EPA TO-15 and TVOC (total volatile organic compounds) via NIOSH 1500. Any samples that were below the limit of detection were assigned a value as the limit of detection divided by the square root of two [27,28,29]. Field blanks (unused canisters) were returned to the laboratory for analysis to ensure that the canisters were not contaminated, and no leaks occurred during shipping. All blank measurements were below the limit of detection, and the vacuum levels remained constant. As such, no adjustments to measurements were made to account for blank concentrations.

Each consumer had a single benzene concentration for the recorded length of their fill-up based on the sampling results. Additionally, each consumer provided the number of times per month they typically fill up their vehicle from the questionnaires. Using the EPA’s most conservative (highest risk) unit risk value for benzene inhalation carcinogenicity of 2.2 × 10^−6^, the excess risk per million people can be calculated following the standard EPA and NIOSH approach in Equation (1) [30,31,32].

(1) $$Excess\;Risk_{per\;1M}=\;1,000,000*\;UR*\;\left(\frac{CA\;*\;ET\;*\;EF\;*\;ED}{AT}\right)$$

From Equation (1), *UR* is the unit risk, *CA* is the benzene concentration, *ET* is the exposure time per day based on the length of time of fill-up, *EF* is the exposure frequency based on fill-ups per year, *ED* is the exposure duration of fifteen years, and *AT* is the averaging time of a lifetime of 70 years. Fifteen years was chosen for the exposure duration based on evidence in the literature that benzene exposures are causative of AML only at an approximate 10–20 year latency and that exposures that occurred more than 20 years prior have no influence on the likelihood of developing leukemia [33,34,35,36,37]. Excess risk values that exceed 1:1,000,000 would be considered an unacceptable risk for consumers, a non-occupational population [31,38].

However, in order to utilize all the collected data and expand the possible combinations of exposure and risk values, a probabilistic Monte Carlo risk approach was utilized as recommended by NIOSH and the EPA for conducting risk assessments [38,39]. Benzene concentrations were log-transformed and parameterized into *N*(x, µ)_Log(Benzene)_. To determine the duration the canister was active, the flow regulator (constant flow rate) and canister had a maximum fill time of six minutes, corresponding to 5 ppm of vacuum decrease per minute of active sampling. Using the initial canister vacuum, subtracting the final canister vacuum, and then dividing by five produced an approximate sampling or fill-up time. Fill times were parametrized into a truncated normal distribution (*Ntrunc*) with a minimum of 0.5 min, maximum of six minutes, and mean and standard deviation based on the calculated fill times per consumer and then converted into *Ntrunc*(x, µ, min = 0.5, max = 6)_Min/Fill_. Fill-ups per month were parameterized into a positive Poisson distribution of count data as *Pois*(λ)_Fill Days/Month_, with λ as the mean of the fill-up counts. The full list of parameters for the consumer risk assessment is provided in Table 1. In order to generate risk values for the consumer population, Equation (2), the probabilistic version of Equation (1), was run 100,000 times with each iteration sampling from the distributions provided in Table 1. Following the resampling using Equation (2) and the values from Table 1, percentiles of risk for the consumer population were calculated from the resulting distribution of excess risk values.

(2) $$Excess\;Risk_{per\;1M}=\;1,000,000*\;UR*\;\left(\frac{\;N{\left(x,\;\sigma \right)}_{Log\left(Benzene\right)}\;*\;\left(\mathrm{Ntrunc}{\left(x,\;\sigma ,\;\min=0.5,\max=6\right)}_{\mathrm{Min}/\mathrm{Fill}}\right)/60\;*\;\left(12*\;\mathrm{Pois}{\left(\lambda \right)}_{\mathrm{Fill}\;\mathrm{Days}/\mathrm{Month}}\right)*\;ED}{AT}\right)$$

Despite sampling for benzene, toluene, ethylbenzene, and xylene, only the benzene concentrations were used in the risk assessment. IARC considers xylene and toluene to be Group 3, or not classifiable to human carcinogenicity, and neither xylene nor toluene have inhalation unit risk values necessary to conduct an inhalation risk assessment [40,41,42,43]. While ethylbenzene is classified as Group 2B, or possibly carcinogenic to humans, the relevant exposure limits provided by OSHA (PEL 100 ppm) and NIOSH (REL 100 ppm), as well as epidemiological studies in ethylbenzene workers, indicate that ethylbenzene’s potential carcinogenic effects would require many orders of magnitude higher levels of exposure than seen in this study to produce excess risk and as such is not considered further here [44,45].

While no direct occupational samples were collected, an occupational exposure scenario was constructed using the near-pump concentrations from the consumer data and a similar probabilistic methodology with appropriate exposure factors for an occupational setting. The consumer exposure concentration distribution of *N*(x, µ)_Log(Benzene)_ was reused directly for the occupational scenario, whereas exposure time and exposure frequency were determined separately for the occupational exposure scenario. Exposure time (length of exposure per day) was assumed to be normally distributed with a mean of seven hours and standard deviation of 0.5 h, *N*(7, 0.5)_Hours/Day_, with the expectation that this is a conservative estimate as it is possible that employees are not actively pumping gasoline an entire workday. Lastly, the exposure frequency (days exposed per year) was drawn from a normal distribution with a mean of 260 days and a standard deviation of ten days, *N*(260, 10)_Work Days/Year_, based on the 260–262 work days in a calendar year and the possibility an employee works more or less based on their personal situation [46]. Furthermore, the occupational risk assessment was conducted with an excess risk management limit of 1:10,000 [31]. The parameters for the occupational risk assessment are shown in Table 2. Again using 100,000 iterations, Equation (3) and the values from Table 2 were used to create the distribution of excess risk values for the occupational scenario.

(3) $$Excess\;Risk_{per\;10K}=\;10,000*\;UR*\;\left(\frac{\;N{\left(x,\;\sigma \right)}_{Log\left(Benzene\right)}\;*\;N{\left(8,\;1\right)}_{Work\;Hours/Day}\;*\;N{\left(260,\;10\right)}_{Work\;Days/Year}\;*\;ED}{AT}\right)$$

After the risk assessments have been completed for both the consumer and occupational exposure scenario, they were contextualized with NATA provided excess cancer risk from ambient benzene concentrations that are provided on the census tract level [16]. However, in order to expand the contextualization for consumers and workers beyond just the specifics of the study population, a probabilistic Monte Carlo approach was used that takes into account the possibility of a consumer living and working in any area of Baltimore City or Baltimore County. Over the course of 100,000 iterations, two random census tract NATA excess risk values were drawn from Baltimore City or Baltimore County, with one being assigned as the home tract with a weight of 0.8 and the other a work tract with a weight of 0.2, based on an approximate 40-h work week. The census tracts were weighted by population for the home tract, so a tract with a higher population is more likely to be selected than a less populated tract. The home and work values were averaged according to their 0.8 and 0.2 weights. Each census tract pair’s averaged NATA excess risk value was then be divided by a random draw from the consumer pumping risk distribution to create a distribution of ratios that compare consumer gasoline pumping risk to ambient risk. Additionally, the same process was conducted for the occupational exposure scenario, where the average NATA excess risk value was divided by a random draw from the occupational risk distribution. Because NATA excess cancer risk is reported as 1:1,000,000, the occupational pumping risk distribution was converted to 1:1,000,000 to allow direct comparison. 

## 3. Results

### 3.1. Consumer Sampling

**Figure 2:**
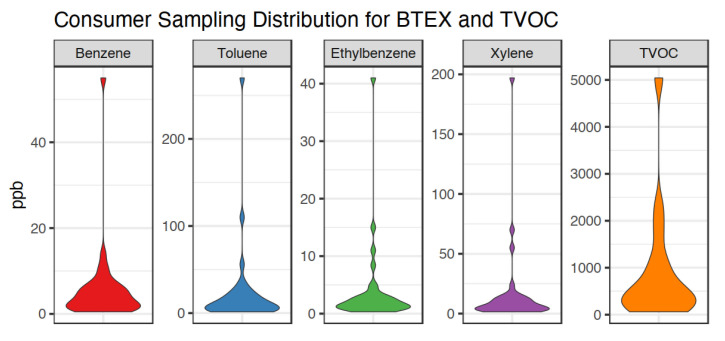
Benzene, toluene, ethylbenzene, xylene, and TVOC concentration distributions from consumer sampling (*n* = 32).

From August 2019 through March 2020, 34 consumer samples were collected. The temperatures during sampling ranged from −1.7 °C to 33.9 °C with a mean of 19.7 °C and a median of 22.5 °C. No relationship between temperature and BTEX or TVOC concentrations was found. Two samples were not used in the exposure and risk assessment process. Consumer 29 had a canister leak in transport and was entirely discarded. Consumer 8′s canister was intact, but the reported sampling results were one to three orders of magnitude above the remaining samples. Consumers 1–10 utilized sampling equipment with inlet valves that had loose gaskets that could require the consumer to touch the inlet orifice to move the gasket out of the way or even return the gasket after it fell out of the equipment entirely. The act of physically manipulating the gasket could have introduced contamination directly into the inlet orifice. Given the potential for gasoline contamination into the sampling apparatus, Consumer 8′s results will not be used for the remainder of the risk assessment. The remainder of samples for Consumers 1–7, 9–10 fell within plausible boundaries of exposure and were retained. Descriptive statistics for the sampling results are presented in Table 3, and violin plots for the sampling distributions for BTEX and TVOC are provided in Figure 2, where plot width indicates the density of samples and the height indicates concentration. 

Of the 32 valid samples, 31 were below the benzene NIOSH REL of 0.1 ppm (100 ppb), and 32 were below the OSHA PEL of 1.0 ppm (1000 ppb) [47]. All samples were below the RELs and PELs for toluene, ethylbenzene, and xylene [40,42,44]. Both the REL and PEL for benzene are 8-hr time weighted averages (TWAs), whereas the samples here are short term task lengths of less than six minutes. The fully parameterized versions of the distributions introduced in Table 1 are presented below in Table 4 based on the results of the personal sampling. Plots of all distributions used in the risk assessments are presented in Appendix A.

### 3.2. Consumer Risk Assessment

**Figure 3:**
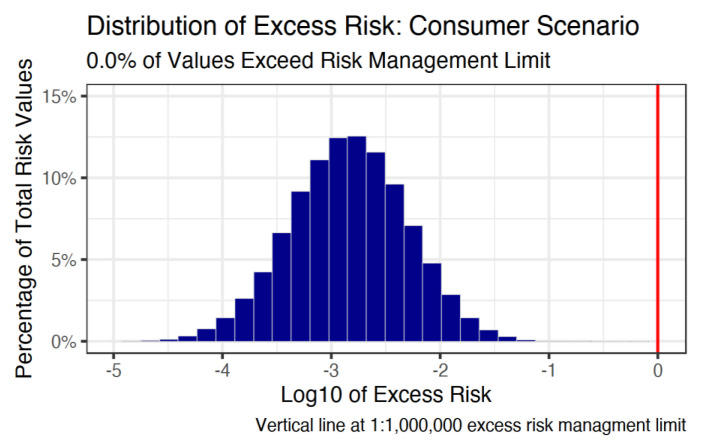
Distribution of excess benzene-related cancer risk from gasoline station pumping for consumers.

Following the probabilistic risk assessment for consumers, zero percent of the 100,000 simulations exceeded the excess risk management level of 1:1,000,000. The full distribution is shown in Figure 3, where 1:1,000,000 is denoted by the vertical line at zero, or the base ten log of one. The 50th percentile of the consumer excess risk distribution was −2.8, the 75th was −2.5, and the 95th was −2.0. Therefore, the 95th percentile of risk was approximately 100 times lower than the excess risk management limit.

### 3.3. Occupational Risk Assessment

**Figure 4:**
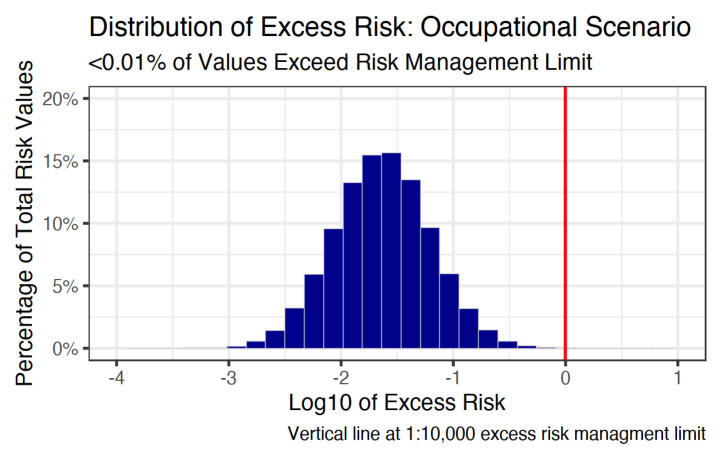
Distribution of the ratio of excess cancer risk from benzene exposure via gasoline pumping to excess cancer risk from ambient benzene exposure for consumers.

The distribution of excess benzene-related cancer risk from gasoline station pumping for the occupational exposure scenario is shown in Figure 4. The distribution exceeded the excess risk management limit of 1:10,000 on less than 0.01 percent of 100,000 trials, with a 50th percentile of −1.6, 75th percentile of −1.3, and a 95th percentile of −0.9. Therefore, the 95th percentile of occupational excess risk is approximately ten times less than the relevant 1:10,000 risk management limit. The excess risk distribution is presented in Figure 4.

### 3.4. National Air Toxics Assessment (NATA) Risk Context

**Figure 5:**
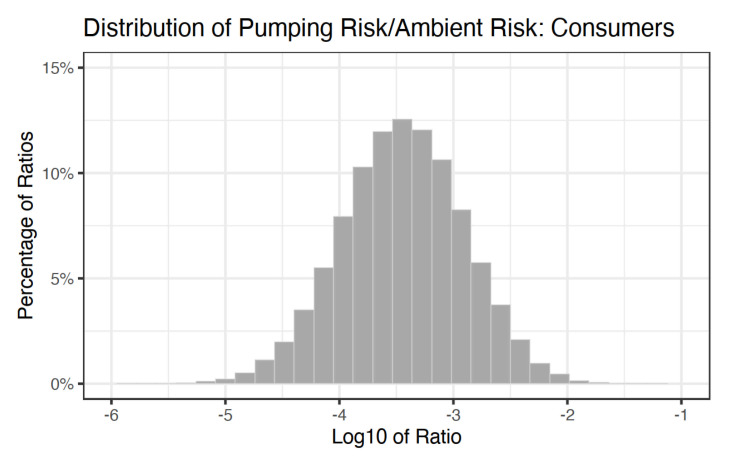
Distribution of the ratio of excess cancer risk from benzene exposure via gasoline pumping to excess cancer risk from ambient benzene exposure for consumer scenario.

**Figure 6:**
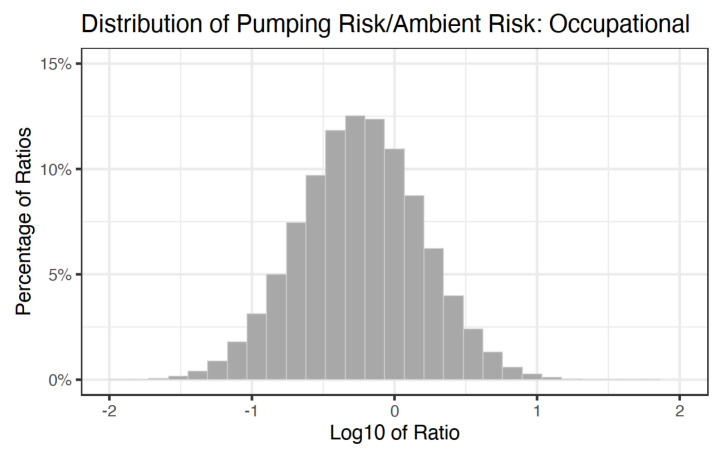
Distribution of the ratio of excess cancer risk from benzene exposure via gasoline pumping to excess cancer risk from ambient benzene exposure for the occupational scenario.

The results of the NATA ratio Monte Carlo are presented in Figure 5 and Figure 6, where both are on the scale of 1:1,000,000. Base ten log ratios greater than zero indicate that the pumping risk for consumer or occupational exposure scenarios exceeds that of the NATA excess risk. For the consumers (Figure 5), zero percent of the simulations exceeded zero. The 50th percentile of the ratio distribution was −3.44, the 75th percentile was −3.08, and the 95th percentile was −2.57, indicating that NATA excess risk was predominantly between two and three orders of magnitude larger than the excess risk from gasoline pumping alone. For the occupational exposure scenario (Figure 6), the 50th percentile was −0.24, the 75th percentile was 0.05, and the 95th percentile was 0.47. Based on the increased exposure duration and frequency, the log base ten ratio distribution for the occupational exposure scenarios exceeded zero on 28.9% of the simulations. 

## 4. Discussion

Millions of individuals per day are exposed to benzene, a known carcinogen, at commercial gasoline stations. In the United States, the most recent comprehensive evaluation of these exposures was conducted in the 1980s [18]. Previous approaches used inconsistent or out of date sampling methodologies and were conducted via simulation studies. In addition, the results were often poorly documented and do not necessarily hold relevance based on the changes to fuel and fuel delivery technology [5,6,7,48]. To address these data gaps and scientific challenges, we implemented a novel self-sampling protocol that allowed consumers to perform their fill-ups as normally as possible, while collecting high-quality exposure data. The strengths of this protocol were that the consumers were likely to fill-up at a gas station they normally use, at a usual time, and in a more standard manner compared with a situation where the consumers were directed to a set study site and observed. The intention was to capture the variability of possible exposure concentrations, and the self-sampling protocol was employed for that reason. Further, using whole air canisters allows for the possibility of measuring a greater number of compounds than single compound methods.

The exposure results for the consumers showed that 32 of the 33 viable samples for benzene were below the NIOSH Recommended Exposure Level of 100 ppb and 33 were below the OSHA Permissible Exposure Level of 1 ppm, despite expected contamination of one sample. While these are occupational standards, the REL is intended to be generally health-protective [49]. Therefore, the consumer samples are less than six-minute exposures at concentrations that NIOSH deems health-protective for eight hours of exposure. Further, consumer benzene exposures from the 1970s–1990s in the United States ranged between approximately 100 ppb and 1000 ppb, whereas the geometric mean exposure from this study was 3.24 ppb [18]. 

In terms of the risk assessment, zero percent of the consumer risk distribution exceeded the 1:1,000,000 excess risk management limit. The occupational distribution had 0.006 percent of the risk values exceed the 1:10,000 excess risk management limit. These risk values for both the consumers and the occupational workers indicate that, when considering strictly pumping gasoline at commercial gasoline stations into automobiles, there is not an unacceptable cancer risk. It is important to note that these risk values do not take into account additional hazardous exposures that are possible at gasoline stations, particularly for an occupational cohort, such as sustained elevated PM_2.5_ exposures from traffic or diesel exhaust fumes. Additionally, this risk assessment is explicitly only for exposures to benzene related to commercial gasoline station fill-ups and does not include other potential sources, such as smoking cigarettes [32]. 

When contextualized with excess cancer risk from Baltimore City and Baltimore County ambient benzene concentrations (NATA), the consumer risk distribution did not exceed the NATA values, whereas approximately 29 percent of the occupational excess risk distribution did exceed the NATA values, indicating that more cancer risk was due to occupational exposure than from ambient exposures. However, Baltimore City (mean = 4.4/1M, std = 0.31/1M) and Baltimore County (mean = 3.76/1M, std = 0.39/1M) are both in the 95th percentile of NATA excess risk from benzene nationwide. Therefore, in counties with lower ambient benzene exposure, gasoline pumping could make up a larger percentage of an individual’s total excess benzene risk than what was presented in Figure 5a,b. Additionally, the lack of spatial data associated with the gas stations (proximity to roadways, traffic patterns, etc.) does not allow for further characterization of station-specific exposures.

The two main limitations of the exposure and risk assessment are the difficulty in verifying that the sampling protocol was followed by the participants and the likely low variability in sampling locations, population, and season. It is possible that additional exposure data outside of the existing distributions would be captured with additional multi-state and multi-season sampling. Further, while significant dermal exposures are not anticipated due to lack of consistent gasoline and skin contact, no dermal exposures were evaluated in this study [50,51]. Finally, COVID-19 research shutdowns limited our sample size to 34 individuals sampled primarily in the fall and winter, with only a few in late summer and zero in the spring. 

## 5. Conclusions

Based on the results of the exposure assessment and the risk assessment, excess cancer risks from benzene exposures due to fuel pumping are low for both consumers and workers. In the context of Baltimore and other urban areas where excess cancer risk from benzene in ambient air is higher than the 1:1,000,000 excess risk management limit for the general population, consumer risks from re-fueling are very low. However, the upper 29 percent of the excess risk distribution for the worker scenario was equivalent, or slightly higher than the ambient benzene risk, but still below the NIOSH risk management limit. Additionally, the use of a novel self-sampling protocol for consumers allowed for a unique exposure assessment on an understudied population that previously relied entirely on simulation studies. The use of whole air sampling means that the protocol can be reused for a range of chemical exposures of concern and could easily be extended to a longer duration task. 

## Figures and Tables

**Table 1:** Parameters for probabilistic consumer risk assessment.

Data	Variable	Value
Benzene Concentration	*CA*	*N*(x, µ)_Log(Benzene)_
Exposure Time	*ET*	*Ntrunc*(x, µ, min = 0.5, max = 6)_Min/Fill_
Exposure Frequency	*EF*	*Pois*(λ)_Fill Days/Month_
Exposure Duration	*ED*	15 years
Averaging Time	*AT*	70 years
Unit Risk	*UR*	2.2 × 10^−6^

**Table 2:** Parameters for probabilistic occupational risk assessment.

Data	Variable	Value
Benzene Concentration	*CA*	*N*(x, µ)_Log(Benzene)_
Exposure Time	*ET*	*N*(7, 0.5)_Work Hours/Day_
Exposure Frequency	*EF*	*N*(260, 10)_Workdays/Year_
Exposure Duration	*ED*	15 years
Averaging Time	*AT*	70 years
Unit Risk	*UR*	2.2 × 10^−6^

**Table 3:** Descriptive statistics for personal sampling results.

Compound	Geo. Mean (ppb)	Geo. SD	Min (ppb)	Max (ppb)
Benzene	3.24	2.72	0.49	55
Toluene	9.50	3.50	1.50	270
Ethylbenzene	1.99	2.80	0.32	41
Xylene	7.32	3.01	1.50	197
TVOC	487.00	3.06	61.04	5042

**Table 4:** Fully parametrized distributions for probabilistic consumer risk assessment.

Data	Variable	Value
Benzene Concentration	*CA*	*N*(−5.73, 0.98)_Log(Benzene)_
Exposure Time	*ET*	*Ntrunc*(3.08, 1.56, min = 0.5, max = 6)_Min/Fill_
Exposure Frequency	*EF*	*Pois*(2)_Fill Days/Month_

**Figure A1:**
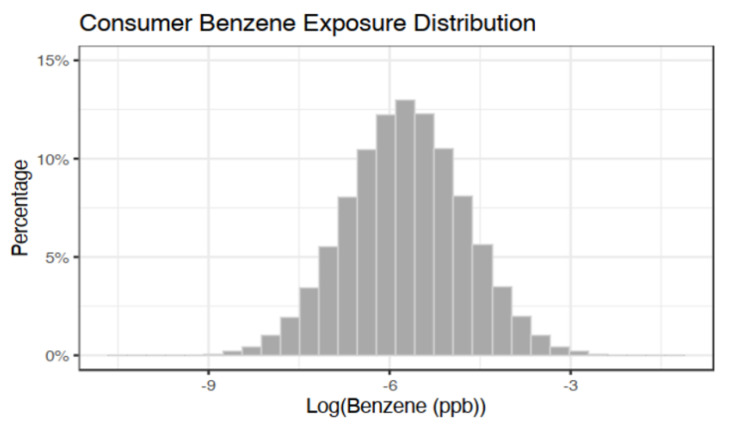
Distribution of consumer benzene exposures on log-ppb scale.

**Figure A2:**
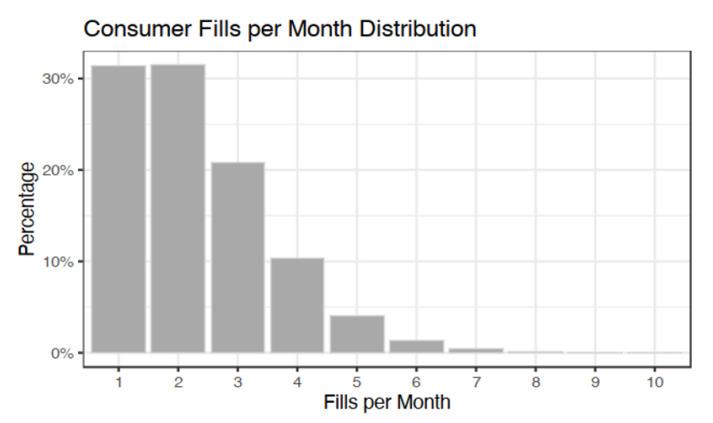
Distribution of consumer fill events per month.

**Figure A3:**
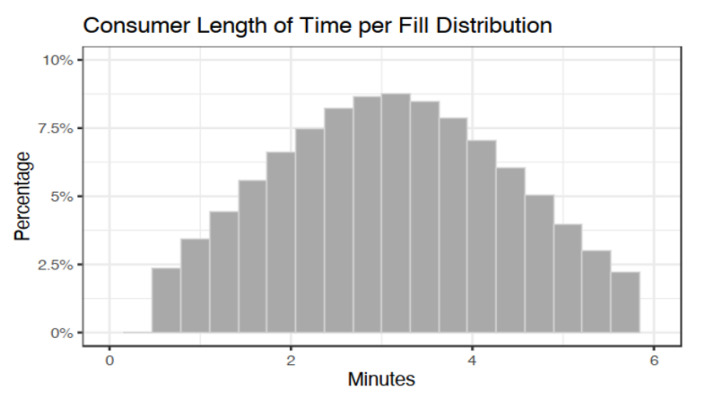
Distribution of consumer fill event durations.

**Figure A4:**
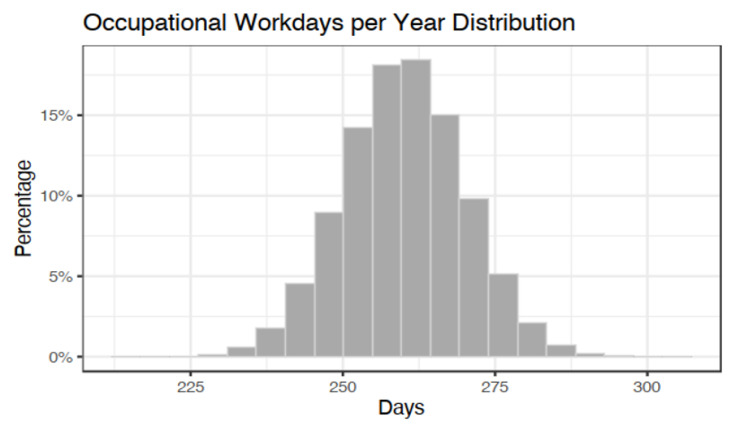
Distribution of occupational days exposed per year.

**Figure A5:**
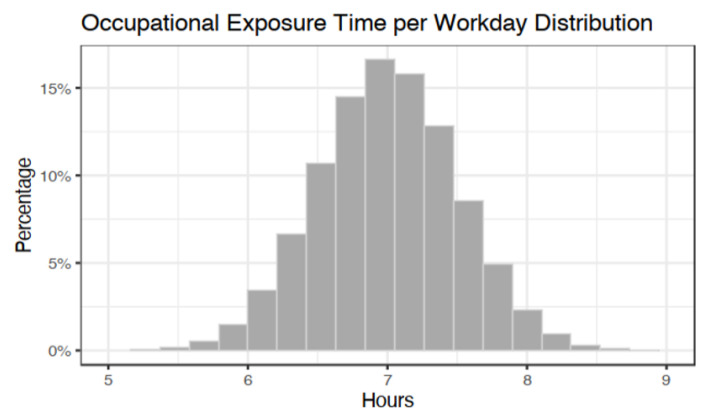
Distribution of occupational hours exposed per workday.

## Data Availability

The de-identified data will be available on the CARTEEH Data Hub following publication.

## Appendix A

The following figures show the distributions used in the consumer (Table 1) and occupational (Table 2) risk assessments. These distributions were based on sampling information, professional judgement, and the literature [44].
